# The Role of Peanuts and Tree Nuts in Improving Diet and Sleep Quality: A Pilot Study and Literature Review

**DOI:** 10.3390/nu18040579

**Published:** 2026-02-10

**Authors:** Alyssa Tindall, Mihaela C. Kissell

**Affiliations:** 1 Department of Health Sciences, Ursinus College, Collegeville, PA 19426, USA; 2 Healthy Mothers Healthy Babies Consortium, Micronutrient Forum, Washington, DC 20005, USA

**Keywords:** nuts, tree nuts, peanuts, sleep, diet quality

## Abstract

**Background**: Peanuts and tree nuts are nutrient-dense foods associated with improved diet quality and reduced chronic disease risk. Diet quality and sleep are interrelated, but the relationship between nut consumption and sleep quality remains understudied, particularly among young adults. **Objective**: This study examined peanut and tree nut consumption, diet quality, and sleep quality in undergraduate students. Existing clinical trials on nut intake and sleep outcomes in healthy adults were reviewed. **Methods**: A pilot study recruited 46 undergraduates to complete three 24 h dietary recalls and self-report sleep quality. Recall days were categorized as containing nuts or no nuts. Diet quality was assessed using the Healthy Eating Index-2020 (HEI). A literature search of PubMed identified human clinical trials testing nut intake with sleep-related outcomes. **Results**: Sixteen percent of the 139 recall days contained nuts. Mean HEI scores were greater on days that contained nuts (64.9 ± 2.3) versus nut-free days (45.4 ± 1.1; *p* < 0.0001). Scores for total fruit, whole fruit, total protein, sea and plant protein, sodium, and refined grains were greater on nut-containing days (*p* < 0.05 for all). Participants reported better sleep on days following nut consumption (*p* = 0.04). From the literature search, four randomized controlled trials (RCTs) were identified with results varying by nut type, dosage, timing, and participants. **Conclusions**: The positive association observed in this pilot study between nut intake and improved diet quality, along with a modest link to better sleep quality, suggests that incorporating nuts regularly into the diet may help enhance overall dietary habits and contribute to improved sleep. The present trials suggest nut intake may improve sleep quality, but significant heterogeneity highlights the need for RCTs with objective sleep outcomes.

## 1. Introduction

Peanuts and tree nuts have a favorable nutrient profile and contain unsaturated fatty acids, protein, vitamins, minerals, bioactive compounds, and fiber [1,2]. Consumption of peanuts and tree nuts improves diet quality [3,4,5,6,7,8,9]. The Global Burden of Disease Study includes data from almost 200 countries and cites low nut and seed consumption among the leading risk factors for chronic disease [10]. The American Heart Association lists diet and sleep among “Life’s Essential 8”, which are vital measures for improving and maintaining cardiovascular health. Nut consumption as part of a healthy dietary pattern could reduce the risk of chronic disease on a global scale.

Childhood lays the foundation for creating healthy habits. However, young adulthood is a particularly pivotal time for individuals to establish healthy habits that can be maintained into adulthood. For many undergraduate college students, leaving home for college or university is the first time complete control can be asserted over both diet and sleep behaviors. Unfortunately, undergraduate college students have both poor diets and sleep habits [11,12].

Short sleep duration can lead to cravings for ultra-processed foods and increase overall energy intake. However, some foods promote better sleep quality, such as milk [13] and omega-3 fatty acids [14]. The correlation between diet quality and other lifestyle factors, such as sleep, emphasizes the importance of understanding if nut intake is associated with both diet and sleep quality. The objective of this pilot study is to examine the relation between peanuts and tree nuts, diet quality, and sleep quality among undergraduate college students. The purpose of this literature review is to describe associations between nut intake and sleep quality in healthy adults.

## 2. Methods

### 2.1. Pilot Study Protocol

This pilot study was conducted from August 2024 to May 2025. Participants were recruited through an undergraduate nutrition class and educated on the Automated Self-Administered 24 h Dietary Assessment Tool (National Cancer Institute, Bethesda, MD, USA) use [15]. The purpose of this study was to examine dietary quality amongst college students, and therefore, nut consumption was not randomized. To assess intake, each participant was provided with a username and password and asked to complete three 24 h diet recalls over the course of four weeks using ASA24. Participants were asked to capture at least one weekend day. After they recorded their 24 h diet recall via the ASA24 website, participants were also asked to answer questions about the quality of their sleep. The ASA24 Sleep Module contains 10 unique questions that address dimensions of sleep health (including sleep timing, efficiency, duration, quality, regularity, and alertness) and were administered immediately after each recall day assessment. Two questions address sleep quality: “how well did you sleep last night?” (answers: 1 = Very good; 2 = Good; 3 = Fair; 4 = Poorly; 5 = Very poorly; removed: 77,777 = I prefer not to answer; 99,999 = I don’t know) and “how did you feel when you woke up today?” (1 = Refreshed; 2 = Somewhat refreshed; 3 = Tired; removed: 77,777 = I prefer not to answer; 99,999 = I don’t know) [16]. Demographic data was collected via Qualtrics. This study was deemed exempt by the Ursinus College Institutional Review Board. Participants were asked to confirm that their data could be used as part of the pilot study through the demographic questionnaire.

Stata software (18.5, StataCorp LLC., College Station, TX, USA) was used for summary statistics, Health Eating Index-2020 (HEI) calculation, and statistical analyses. Recall days were evaluated by a registered dietitian (AT) for plausibility, and any participant who had fewer than two recall days was removed. Each recall day was classified as ‘nuts consumed’ if the participant reported consuming peanuts, peanut butter, tree nuts, or tree nut butter (e.g., almond butter), or ‘no nuts consumed’ if the aforementioned were absent. Days with nut-based beverages (plant-based milks such as almond milk) did not count towards ‘nuts consumed’ as they differ nutritionally from whole nuts and nut butters (purees). Per the non-randomized design, the recall days were not averaged for each participant since participant consumption of nuts was mixed over recall days. Further, to compare individual recall days to self-reported sleep quality questions that referenced the prior night’s sleep, diet recall days could not be averaged. HEI and corresponding sub-components were used to measure diet quality and calculated for each recall day. The overall HEI is made up of 13 components that reflect the different food groups and key recommendations to be consumed in adequacy and moderation in the Dietary Guidelines for Americans, 2020–2025. Each sub-component is scored out of 5 or 10 points (a max score indicates recommendations were met), and total diet quality is scored out of 100 points [17].

### 2.2. Literature Search Protocol

PubMed search terms included the following: “nuts AND sleep”, “almonds AND sleep”, “Brazil nuts AND sleep”, “cashews AND sleep”, “hazelnuts AND sleep”, “macadamia nuts AND sleep”, “peanuts AND sleep”, “pecans AND sleep”, “pine nuts AND sleep”, “pistachios AND sleep”, “walnuts AND sleep”. Studies were included based on the inclusion criteria of human clinical trials with sleep-related outcomes, such as sleep efficiency. Exclusion criteria included articles that were not published in English. The searches were conducted from inception until August 2025. Abstracts were further reviewed by the authors (AT and MK) for the following criteria: ‘normal’ sleepers, whole nuts or nut puree as intervention, and sleep as an outcome.

## 3. Results

### 3.1. Pilot Study Findings

**Figure 1:**
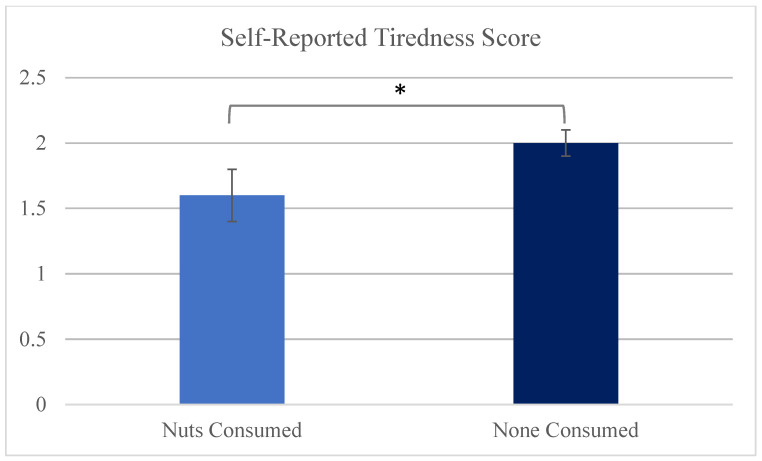
Self-reported sleep quality reported as tiredness score by nuts consumed vs. none consumed. * indicates statistical significance (*p* < 0.05).

This was a convenience sample that consisted of 46 participants (Table 1) who tracked 139 recall days (2–5 recall days per participant). Only 14% of recall days included nuts. Thirty-two participants (120 recall days) did not have a recall day that contained nuts. Due to the non-randomized design, differences in participants who consumed nuts versus those who did not consume nuts could not be tested, as some participants had both recall days that contained nuts and recall days that did not contain nuts. Diet quality was significantly greater on days that contained nuts (19 days; 14 participants; Table 2) compared to those that did not contain nuts (*p* < 0.0001). The diet quality sub-components that contributed to this difference were: total fruit, whole fruit, total protein, sea and plant protein, sodium, and refined grains (*p* for all <0.05; Table 2). Better sleep quality was reported (feeling significantly more refreshed) following days when nuts were consumed compared to days when no nuts were consumed (*p* = 0.04; Figure 1). Given the limited number of days containing nuts, a within-participant comparison was completed of days in which nuts were consumed versus days in which nuts were not consumed (*n* = 12). Sleep variable scores were averaged for participants who had two days reported with no nuts or two days with nuts. There were no significant differences in self-reported sleep variables between days when nuts were consumed versus when no nuts were consumed among participants.

Subjective sleep quality was assessed via the Automated Self-Administered 24 h Dietary Assessment Tool sleep question. The following questions related to sleep quality were answered by participants: “How did you feel when you woke up today?” 1 = Refreshed; 2 = Somewhat refreshed; 3 = Tired; (removed: = I prefer not to answer; 99,999 = I don’t know) [tiredness; above graph]; “how well did you sleep last night?” (answers: 1 = Very good; 2 = Good; 3 = Fair; 4 = Poorly; 5 = Very poorly; removed: 77,777 = I prefer not to answer; 99,999 = I don’t know). The latter was not statistically significant.

### 3.2. Literature Search Findings

Forty-four articles were identified as part of the literature search. Four were included in the review (Table 3). The other forty were excluded for the following reasons: four duplicated articles, three for ‘abnormal’ sleepers (e.g., social jetlag, narcolepsy-like sleepers, Dravet syndrome), nine for nut oil testing (whole nut studies or pureed nut studies were included), two protocol papers, eight for missing sleep as an outcome, fourteen for missing nuts as an intervention.

Four supplementation trials were identified that tested the impact of almonds, peanuts (peanut butter), and walnuts on sleep quality. Significant variability exists between trials, including the treatment/dose, outcome measurement, participants, and length of follow-up. The dose ranged from approximately 0.5 to 2 oz of nuts. All investigators randomly assigned participants to groups; however, only one of the trials reported providing a dietary intervention to the control group. Two studies collected dietary data from participants. Participants from all trials were overall healthy, but sex was unbalanced in all four trials. The length of follow-up ranged from six weeks to one year. Per the search requirements, all trials included sleep quality as an outcome, but authors used different measurement tools (Actigraphy, Pittsburgh Sleep Quality Index, Spiegel Questionnaire, Epworth Sleepiness Scale).

Brown et al. [19] and Oberther et al. [20] both reported null results for nut intake on sleep quality. Despite providing a higher dose and longer follow-up, there were no differences between the intervention and control groups. However, both trials included males and females with a younger average participant age (mid-thirties) compared to Kamoun et al. [21], which included only older adults who identified as male. The former two trials used differing protocols for nut consumption: Brown et al. encouraged participants to consume nuts or a control snack at the first snacking occurrence, compared to Oberther et al., who asked participants to consume peanut butter two hours prior to bedtime. Further, Oberther et al. only required participants to consume the intervention (peanut butter) five nights per week compared to both other trials, which asked participants to consume the intervention daily.

Kamoun et al. [21] and Zerón-Rugerio et al. [22] both reported improvements in sleep quality with walnut consumption. Kamoun et al. reported that 15 g of walnuts in addition to exercise training improved sleep quality compared to the training-only control group. Both groups experienced improvements in sleep quality from baseline, but the walnut group reported significantly greater improvement than the control. This trial was different from the others as it only recruited older adults who identified as male, concurrently physically trained participants, provided the lowest dose of nuts (0.5 oz/day), asked participants to consume nuts in the morning (10 AM), and utilized the Spiegel Questionnaire. Zerón-Rugerio et al. employed a larger walnut dose of 40 g and instructed participants to consume nuts with dinner. This study included younger adults who predominantly identified as female. The authors reported improvements in sleep quality, such as sleep latency, and found greater urinary melatonin metabolites in the walnut intervention versus the control. The findings from this trial, together with the aforementioned, suggest the impact of nut consumption on sleep quality may depend on the nut type and timing of nut intake; participant characteristics could also play a role.

## 4. Discussion

The present pilot study provides evidence that the population of interest (university college-aged students) can provide high-quality dietary data with limited oversight, and nut consumption is unexpectedly low in this sample of university students. The majority of reported days did not contain nuts (86%), yet on the days nuts were consumed, diet quality and sleep quality were significantly improved. However, when within-participant comparisons (*n* = 12) of nut-containing days vs. non-nut-containing days were tested, there were no significant differences in sleep quality. The pilot study illustrates that nut consumption improves diet quality and may impact sleep. However, a larger RCT is required to confirm these pilot findings. Mean diet quality on days nuts were consumed (total HEI score: 65) was above the national average for this age group (scores for 2–18 and 19–59 years, respectively: 54–57 [23]) compared to days no nuts were consumed (score: 45), which was below the national average. The literature search identified four RCTs that fit the search criteria and suggested a weak association between nut consumption and sleep; two of the four trials examined reported significant improvements in sleep quality. The heterogeneity and limited number of trials emphasize the need for a larger, well-designed study to examine the association between nuts and sleep. In comparison with the present pilot study, which included a younger population, an even split between male and female sex, and no nutritional guidance or supplemental nuts, the literature review suggests the impact of nut intake on sleep quality may depend on the nut type and timing of consumption; participant age and sex could also play a role.

In the pilot study, the greater HEI scores on nut-containing days align with current evidence and recommendations that peanuts and tree nuts improve diet quality [3,4,5,6,7,8,9]. Total diet quality, total protein, and sea and plant protein scores were greater on the nut-containing days, which was expected with increased nut intake. However, refined grain scores were also greater on the nut days, yet there were no differences in fat scores. It is possible that days with nuts may reflect overall healthier dietary patterns, not necessarily attributable to nut consumption. Nonetheless, incorporating nuts into a dietary pattern may be a simple strategy to improve diet quality. For example, nuts are a key component of the Mediterranean diet, which is a dietary pattern recommended by the 2020–2025 Dietary Guidelines for Americans [18], and may improve sleep quality. The Mediterranean diet emphasizes legumes, fruits, whole grains, and nuts high in melatonin [24]. The melatonin content of nuts, together with other beneficial features of the Mediterranean diet, such as antioxidant, anti-inflammatory, and anti-neurodegeneration properties, may explain the more adequate sleep achieved in those who adhere to a Mediterranean diet [25].

The present pilot study is limited by the number of days nuts were consumed and the metrics in which sleep was assessed. Yet the possible association between nut intake and sleep reported aligns with the potential of nuts’ nutrition profile contributing to better sleep among otherwise healthy adults. Micronutrients linked with sleep include iron, zinc, magnesium, copper, vitamin K, and B12 [9]. Data from a nationally representative sample of adults identified a significant association between short sleep duration and the percentage of the population with intakes below the estimated average requirement for micronutrients, including copper, iron, magnesium, zinc, folate, and vitamins A, B2, C, and K [26]. Tree nuts are good sources of several of these nutrients, such as copper, magnesium, iron, zinc, folate, niacin, and vitamins C and B2 [27]. Cashews and almonds contain the greatest amount of iron and zinc compared to the other tree nuts [2], which have been positively associated with sleep duration [28]. Further, poor iron status has been linked with poor sleep, primarily in infancy [29]. This evidence indicates that iron may have a role in sleep, and maintaining an adequate iron status may benefit or even improve sleep. Magnesium is among the most studied sleep-related micronutrients. Walnuts and almonds contain the greatest magnesium compared to the other tree nuts [2]. Based on a systematic review of observational evidence, greater magnesium intake is linked with sleep quality (daytime sleepiness), especially in females [2]. Limited evidence from human and animal studies points out that magnesium deficiency may lead to poor sleep quality, but suggests a positive effect of magnesium supplementation on sleep [30]. Mechanistically, magnesium can increase melatonin and renin and reduce cortisol levels, therefore potentially improving sleep quality [31,32]. Magnesium is also a cofactor of many enzymes (>300) that regulate biological reactions [33], including circadian rhythm [34]. Data that describes mechanisms of micronutrient involvement in sleep neurobiology are limited, but suggest micronutrients are involved in the synthesis and transport of neurotransmitters that regulate sleep [9].

Nut intake can impact hemodynamics and, therefore, may also affect sleep. Blood pressure drops below daytime levels as part of a normal sleep pattern. Cardiovascular risk increases in the absence of overnight blood pressure dipping. Sauder et al. [35] conducted a randomized, crossover, controlled-feeding study in which participants were provided a moderate-fat diet containing pistachios (20% of total energy) compared to a low-fat control diet for four weeks. The pistachio diet significantly improved systemic hemodynamics, heart rate variability, and systolic ambulatory blood pressure was significantly reduced by 3.5 ± 2.2 mm Hg (*p* = 0.046) following the pistachio diet, with the greatest reduction observed during sleep (−5.7 ± 2.6 mm Hg, *p* = 0.052). This finding can significantly impact cardiovascular risk. For example, a meta-analysis of 48 randomized trials reported that a 5 mm Hg reduction in systolic blood pressure reduced the risk of major cardiovascular events by about 10% [36]. Although this study did not examine sleep quality, the dipping pattern reported with pistachio consumption suggests participants may also have experienced normal or improved sleep.

The timing of nut ingestion may impact sleep through the present bioactive components. For example, melatonin is a hormone involved in sleep regulation and can be obtained exogenously or synthesized endogenously. Exogenous melatonin can be found in some tree nuts and can decrease sleep onset latency, increase sleep efficiency, and increase total sleep duration. Melatonin administered during the day, when endogenous levels of the hormone are low, can increase sleep efficiency [37]. The sleep improvements Kamoun et al. [21] reported with morning walnut consumption are of interest due to the melatonin and fatty acid profile present in walnuts [38]. Further, Zerón-Rugerio et al. [22] reported that walnut consumption increases urinary melatonin metabolite, 6-sulphatoxymelatonin. Although the melatonin content of walnuts is approximately 100 ng/oz (3.5 ± 1.0 ng/g) and much lower than supplemental doses of melatonin (typical dose 1–5 mg), the timing of intake may have a greater impact on sleep outcomes. Kamoun et al. [21] reported better sleep quality with 15 g walnuts per day, which participants were recommended to consume in the morning. In contrast, Brown et al. [19] recommended participants consume almonds (or a control snack) at the first snack of the day, and the authors did not observe a significant change in sleep quality; however, the timing of the first snack is not reported. Oberther et al. [20] instructed participants to consume the peanut butter intervention before bedtime and reported no significant improvements in sleep quality. The melatonin present in walnuts, in combination with the timing at which the walnuts were consumed, may be key components in affecting sleep quality. However, the varying doses and measurement tools make it difficult to understand the role nut consumption may have on sleep quality.

Together with the present pilot study, which used subjective sleep measurement (ASA24 sleep module), each of the four trials reviewed not only tested different nuts but also used different sleep measurement tools. The heterogeneous designs could impact the individual trial findings and clinical implications. Further, certain components of sleep measurements may have a greater impact on health. For example, an umbrella review of 69 meta-analyses found a dose–response analysis between sleep duration and health; a 1 h reduction in sleep duration per 24 h was associated with an increased risk by 3–11% of all-cause mortality, heart disease, osteoporosis, stroke, and type 2 diabetes among short sleepers [39]. The trials reviewed measured sleep duration among other metrics, but utilized different methods. Kamoun et al. [21] and Brown et al. [19] both used sleep questionnaires (Pittsburgh Sleep Quality Index [PSQI] and the Spiegel Questionnaire, respectively). The PSQI is a self-report questionnaire used to assess sleep over a one-month interval and examines the following domains: subjective sleep quality, sleep onset latency, sleep duration, sleep efficiency, sleep disturbances, use of sleep medication, and daytime dysfunction. The Spiegel Questionnaire is offered in French, utilizes self-report to measure subjective sleep quality, and asks questions based on the prior night (questions/domains: sleep onset delay, quality of sleep, duration of sleep, nighttime awakenings, dream occurrence, state in the morning). Oberther et al. [20] and Zerón-Rugerio et al. [22] utilized an objective sleep measurement, actigraphy (Actigraph wristwatches), to measure sleep variables over the course of the trials. In Oberther et al. [20], various weeks were selected for comparison of sleep variables, but the rationale for week selection is unclear. However, the authors did not report any group x time effects over the course of the first eight weeks of the trial in regard to the sleep variables (sleep latency, total sleep time, time in bed, sleep efficiency, wake after sleep onset, number of awakenings per night, and time spent awake per night). The substantial variability in sleep quality assessment in each trial makes it challenging to compare sleep quality outcomes across studies.

The variability in participant characteristics between the pilot and four trials may also impact sleep-related findings. Sleep can vary by age and sex [40], which both vary across the trials presented in this article. For example, a systematic review and meta-analysis of over a million individuals across the lifespan reported that teenagers had shorter total sleep time compared to adults who primarily suffered from poor sleep quality and insomnia symptoms. Furthermore, a systematic review that included studies utilizing the gold standard of sleep measurement, polysomnography, indicated that for each decade of age, total sleep time and sleep efficiency decreased while wake after sleep onset, sleep onset latency, arousal index, and periodic limb movement index increased [41,42]. The changes that occur in sleep health over the lifespan may impact the efficacy of nutritional interventions, such as nuts, on sleep quality. For example, total sleep time and sleep efficiency may be more critical metrics among younger populations experiencing challenges related to sleep duration, while wake after sleep onset and arousal index may be more important to assess in older populations that have greater issues with fragmented sleep. Electrical activity of the brain during sleep may also differ between sexes. There is moderate evidence to suggest females have a steeper delta wave slope and males have greater normalized delta power during sleep [43]. These differences may partially explain the heterogeneity in trial results. Future trials should consider that both age and sex could impact the efficacy of nut intake on sleep quality.

The present pilot study is not without limitations. A small convenience sample was used, which limits generalizability. Furthermore, participants were not assigned to consume nuts or not consume nuts, and only 16% of recall days contained nuts. Due to the non-randomization, within-participant recall days were mixed in terms of nut consumption. Therefore, between-participant differences related to nut consumption could not be tested. An RCT with objective sleep measures would provide a better understanding of the relation between nut intake and sleep quality. The literature review also indicated a need for further examination of this topic, given that there was substantial heterogeneity between trials regarding design, dose, timing, and population. Only four trials were identified that tested the impact of nut consumption on sleep. Together, this pilot study and literature review provide a foundation for understanding the effect of peanut and tree nut intake on sleep quality; however, well-designed trials are needed to confirm current knowledge and understand underlying mechanisms. Once additional RCTs are conducted, a scoping review or systematic review could provide greater clarity on this topic.

## 5. Conclusions

In conclusion, the expected low diet quality among college undergraduate students indicates a need for improving students’ dietary habits. However, the low intake of peanuts and tree nuts was unexpected and should be taken into account in the design of future trials within this population. The favorable correlation between nut intake, diet, and sleep quality in the pilot trial emphasizes the potential for improving dietary patterns and sleep health through regular nut consumption, but is limited by the study design, the number of days participants consumed nuts, and the subjective sleep quality outcome. The present pilot study, together with the literature review, suggests potential for nut intake to improve sleep quality; however, the significant heterogeneity between trials highlights the need for RCTs with objective sleep outcomes.

## Figures and Tables

**Table 1:** Background characteristics.

Variable	*n*
Sex	
Female	26
Male	20
Athlete status	
Yes	31
No	15
College year	
Sophomore	3
Junior	27
Senior	16
Age	
18–19	3
20–21	38
22–23	5

**Table 2:** Dietary and sleep differences between days containing nuts vs. no nuts.

Variable	Nuts Consumed (Mean ± SD)	None Consumed	*p*-Value
Total Healthy Eating Index	64.9 ± 2.3	45.4 ± 1.1	<0.0001
Total Fruit	2.7 ± 1.6	1.5 ± 2.0	0.008
Whole Fruit	3.8 ± 1.7	1.5 ± 2.1	<0.0001
Total Protein	4.8 ± 0.4	4.3 ± 1.2	0.0004
Sea and Plant Protein	4.4 ± 0.8	1.5 ± 1.9	<0.0001
Sodium	6.3 ± 3.3	3.4 ± 3.4	0.002
Refined Grains	8.5 ± 2.5	4.5 ± 3.9	<0.0001
Whole Grains	3.4 ± 3.3	2.1 ± 3.0	0.12
Total Dairy	6.8 ± 3.4	5.9 ± 3.0	0.27
Total Vegetables	3.4 ± 1.6	2.7 ± 1.6	0.07
Dark Green Leafy Vegetables	2.8 ± 2.3	1.9 ± 2.2	0.13
Unsaturated Fatty Acids	4.1 ± 3.7	4.1 ± 3.3	0.97
Added Sugars	8.4 ± 1.9	7.5 ± 2.8	0.09
Saturated Fatty Acids	5.6 ± 3.7	4.6 ± 3.5	0.30

Total Healthy Eating Index (HEI) and corresponding subcomponents were calculated for each recall day. The overall HEI-2020 is made up of 13 components that reflect the different food groups and key recommendations to be consumed in adequacy and moderation in the Dietary Guidelines for Americans, 2020–2025 [18]. Each subcomponent is scored out of 5 or 10 points (a max score indicates recommendations were met), and total diet quality is scored out of 100 points. Walnut days, *n* = 19; non-walnut days, *n* = 120.

**Table 3:** Summary of RCTs testing the effects of nut consumption on sleep quality.

References	Treatment	Dose	Study Design	Control Treatment	Subjects	*n*	Male (%)	Results	Length of Follow-Up
Brown et al. 2023 [19]	Almonds	42.5 g	RCT	Cracker biscuits	Healthy adults	136	26	No differences in sleep quality	12 months
Oberther et al. 2024 [20]	Peanut Butter	32 g	RCT	Ad libitum diet	Firefighters	40	98	No differences in sleep quality	1.75 months
Kamoun et al. 2024 [21]	Walnuts	15 g	RCT	Ad libitum diet	Adults ≥ 65 years	20	100	Sleep quality improved in the walnut group vs. the control	1.5 months
Zerón-Rugerio et al. 2025 [22]	Walnuts	40 g	RCT	Ad libitum diet	Young adults	76	14	Sleep quality improved in walnut vs. control	8 weeks

Abbreviations: RCT = randomized controlled trial.

## Data Availability

Data generated for this pilot study cannot be shared publicly or upon request, as participants only consented to their data being used in the pilot study at Ursinus College.
